# Serum Amyloid Alpha Is Downregulated in Peripheral Tissues of Parkinson’s Disease Patients

**DOI:** 10.3389/fnins.2019.00013

**Published:** 2019-01-29

**Authors:** Lille Kurvits, Ene Reimann, Liis Kadastik-Eerme, Laura Truu, Külli Kingo, Triin Erm, Sulev Kõks, Pille Taba, Anu Planken

**Affiliations:** ^1^Department of Neurology and Neurosurgery, University of Tartu, Tartu, Estonia; ^2^Department of Neurology, Charité – Universitätsmedizin Berlin, Berlin, Germany; ^3^Institute of Pathophysiology, University of Tartu, Tartu, Estonia; ^4^Laboratory of Bioenergetics, National Institute of Chemical Physics and Biophysics, Tallinn, Estonia; ^5^Department of Dermatology, University of Tartu, Tartu, Estonia; ^6^Dermatology Clinic, Tartu University Hospital, Tartu, Estonia; ^7^Department of Pathology, Tartu University Hospital, Tartu, Estonia; ^8^Centre for Comparative Genomics, Murdoch University, Perth, WA, Australia; ^9^Perron Institute for Neurological and Translational Science, University of Western Australia, Perth, WA, Australia; ^10^Oncology and Haematology Clinic, North-Estonian Medical Centre, Tallinn, Estonia

**Keywords:** neurodegenarative disease, serum amyloid A (SAA), Parkinson’s disease (PD), intrinsically disordered protein (IDP), skin biopsy

## Abstract

We report the changed levels of serum amyloid alpha, an immunologically active protein, in Parkinson’s disease (PD) patients’ peripheral tissues. We have previously shown that *Saa-1* and *-2* (serum amyloid alpha-1,-2, genes) were among the top downregulated genes in PD patients’ skin, using whole-genome RNA sequencing. In the current study, we characterized the gene and protein expression profiles of skin and blood samples from patients with confirmed PD diagnosis and age/sex matched controls. qRT-PCR analysis of PD skin demonstrated downregulation of *Saa-1* and *-2* genes in PD patients. However, the lowered amount of protein could not be visualized using immunohistochemistry, due to low quantity of SAA (Serum Amyloid Alpha, protein) in skin. *Saa-1* and *-2* expression levels in whole blood were below detection threshold based on RNA sequencing, however significantly lowered protein levels of SAA1/2 in PD patients’ serum were shown with ELISA, implying that SAA is secreted into the blood. These results show that SAA is differentially expressed in the peripheral tissues of PD patients.

## Introduction

Parkinson’s disease (PD) is a neurodegenerative disorder, characterized by motor symptoms, like resting tremor, hypo- and bradykinesia, rigidity and postural instability (Poewe et al., 2017). However, the clinical specter of affected functions is much wider due to the involvement of other central and peripheral organ systems. Pathologic neuronal lesions outside the central nervous system (CNS) have been demonstrated in PD patients (Gelpi et al., 2014), for example characteristic α-synuclein deposits have been found in dermal nerve fibers of PD patients skin (Wang et al., 2013). In addition, pathogenic biomolecular defects of PD occur in non-neuronal peripheral tissues (Braak et al., 2003; Auburger et al., 2012; Teves et al., 2018). PD patients have many dermatologic problems like seborrhoea, seborrheic dermatitis, hyperhidrosis and impaired wound healing (Gregory and Miller, 2015). As to date the affirmative diagnosis of PD can only be made based on the results of post-mortem autopsy, thus there is a great need for detailed understanding of disease biology, as well as for studies investigating novel diagnostic and prognostic biomarkers. Peripheral blood has been widely used in the search for biomarkers in PD (Scherzer et al., 2007; Grünblatt et al., 2010; Borrageiro et al., 2017), however other peripheral tissues, such as skin, are promising models for investigating the pathogenic mechanisms of PD.

Serum amyloid alpha (SAA) is a protein that may be linked to PD due to its many functions in metabolic networks affected in PD. SAA plays a key role in the functioning of the immune system, being involved in inflammation, tumorigenesis, regulating skin homeostasis and accumulating as misfolded AA-amyloid protein (Artl et al., 2000; Urieli-Shoval et al., 2000). SAA1 and SAA2 expression is induced in the liver, which is the major site of synthesis. It is also produced extrahepatically, especially within dermal tissues by keratinocytes (Urieli-Shoval et al., 1998; Upragarin et al., 2005). Different types of cancers occur less frequently in PD patients, however the risk of melanoma is about 2-fold higher than for controls (Devine et al., 2011; Liu et al., 2011). Serum SAA has been shown to be elevated in melanoma patients through all stages of disease and is proposed to be a prognostic cancer biomarker (Findeisen et al., 2009).

The current study was undertaken based on observations from our previous transcriptomic analysis on PD patients’ skin, which demonstrated serum amyloid alpha 1 and 2 genes (*Saa-1,-2)* to be one of the most significantly deregulated genes in PD skin (Planken et al., 2017). It was of interest, whether the changes in RNA levels are constant and transcribed into changed protein levels within different tissues.

## Materials and Methods

### Study Subjects

We enrolled in total 86 PD patients who were diagnosed by neurology-board-certified movement disorders specialists and met the Queen Square Brain Bank diagnostic criteria (Gibb and Lees, 1988; Lees et al., 2009) from Tartu University Hospital. 72 healthy matched controls with no personal history of neurodegenerative diseases were also enrolled. Independent samples of PD patients with comparable age, gender, Movement Disorders Society’s Unified Parkinson’s Disease Rating Scale (MDS-UPDRS, Goetz et al., 2008) and the Hoehn and Yahr Scale (H&Y, Hoehn and Yahr, 1967) were randomly allocated for quantitative real time polymerase chain reaction (qRT-PCR), enzyme-linked immunosorbent assay (ELISA) and Immunohistochemistry. In total, the PD patients had an average age of 71.0 ± 7.8 years, MDS-UPDRS of 52.0 ± 14.0, H&Y of 2.7 ± 0.9 and 85.0% had no family history of PD. Clinical details of the participants are provided in Table 1 (breakdown of clinical details of different groups in Supplementary Table S1). All PD patients received standard medications. As a limitation to our study, the inflammatory status of all subjects is not known, but those tested (4 PD patients) had serum CRP < 1 mg/L and serum cholesterol < 5.2 mmol/L. The study was approved by the local Ethics Committee and an informed consent was obtained from all patients and controls included in the study.

**Table 1:** Demographic characteristics for total PD patients and controls.

Samples	PD qRT-PCR	PD ELISA	PD Immuno-histo	HC qRT-PCR	HC ELISA	HC immuno-histo	*P*
Age at enrolment (years, *SD*)	69.5 ± 7.3	72.1 ± 8.4	72.0 ± 7.2	72.1 ± 7.9	72.7 ± 9.6	71.8 ± 8.6	n.s.
Age of PD onset (years, *SD*)	61.4 ± 8.3	66.5 ± 10.8	65.6 ± 9.2	n/a	n/a	n/a	n.s.
Male gender (*n*, %)	18 (49)	12 (33)	6 (46)	12 (36)	10 (37)	3(25)	n.s.
1st degree relatives with PD (*n*, %)	3 (8.1)	4 (11.4)	0	n/a	n/a	n/a	n.s.
MDS-UPDRS (mean, *SD*)	62.7 ± 24.8	71.8 ± 30.7	65.8 ± 27.1	n/a	n/a	n/a	n.s.
H&Y (mean, *SD*)	2.5 ± 0.8	2.7 ± 1.0	3.1 ± 0.8	n/a	n/a	n/a	n.s.


*There were no significant (n.s.; p > 0.05) differences between independent samples by analysis of variance with post hoc Tukey test.*

### Tissue Sampling and RNA Extraction

Skin punch-biopsies of Ø 4 mm were taken from non-sun-exposed skin from the medial side of upper arm, instantly frozen in liquid nitrogen and stored at –80°C until RNA extraction. Biopsies were homogenized with Precellys24 homogenizer with the Cryolys system (Bertin Technologies). RNeasy Fibrous Tissue Mini Kit (Qiagen) was used for total RNA extraction, according to the manufacturer’s protocol. During the purification on-column DNase I was applied (Qiagen). The RNA quality was assessed using Agilent 2100 Bioanalyzer, the RNA 6000 Nano kit (Agilent Technologies) and the Qubit fluorometer (Life Technologies). The lowest RIN of samples was 6.7.

### Real-Time Quantitative PCR

Blood samples of 37 patients and 33 healthy controls were obtained. Total RNA from skin biopsies was converted to cDNA using random primers and High Capacity cDNA Reverse Transcription Kit with RNase Inhibitor (Applied Biosystems). For duplex qRT-PCR analysis TaqMan Gene Expression Assays were used: VIC (housekeeping gene ActinB) and FAM (gene of interest) probes and TaqMan Gene^®^ Expression Master Mix (Applied Biosystems). The TaqMan^®^ Gene Assay IDs were the following: Hs01060665_g1 (*ActinB*), Hs00761940_s1 (*Saa1*), Hs00754237_s1 (*Saa2*). qRT-PCR was performed using ABI PRISM 7900HT Fast Real-Time PCR System equipment (Applied Biosystems) and the ABI PRISM 7900 SDS 2.2.2 Software. Each reaction was performed in quadruplicate to minimize technical errors. Real-time PCR data for gene of interest was expressed as mean ΔCT value relative to housekeeping gene. ΔCT of controls was subtracted from ΔCT of PD to yield ΔΔCT. Relative expression i.e. fold change was calculated using 2^-ΔCT^-function. The data of studied genes following normal distribution were parametrically tested by unpaired *t*-test.

### Enzyme-Linked Immunosorbent Assay of the Blood Samples

Blood samples of 36 patients and 27 healthy controls were obtained. Serum was extracted from blood samples and rapidly frozen to and stored at –80°C. Stored samples were defrosted and centrifuged at 1500 g at room temperature for 10 min. ELISA kit for human SAA1/2 (Invitrogen Corporation) was used. 200-fold diluted human serum SAA1/2 standard was calibrated to a highly purified *Escherichia coli*-expressed recombinant protein. The sample measurements were performed with Tecan GeniOS Pro luminometer in duplicate and repeated 3 times on separate plates. Optical density (450 nm) readings were used to quantitatively express serum SAA1/2 results. The Human SAA1/2 concentrations for samples and controls were plotted based on the standard curve. Values obtained for serum were multiplied by 200 to correct for the overall dilution. The data for mean concentration followed normal distribution, was plotted on a barplot and parametrically tested by unpaired *t*-test.

### Skin Immunohistochemistry

Immunohistochemistry samples were obtained from 13 PD patients (6 male, 7 female, mean age of 72.0 ± 7.2 years) and 12 controls (6 male, 6 female, mean age of 71.3 ± 7.1 years). The skin biopsies were deparaffinized with 2× 4 min. xylene, 4 min. isopropanol, 2× 4 min. 96% alcohol. Then they were blocked with 3% hydrogen peroxide for 7 min, and processed with proteinase K for 5 min. The slides were incubated with primary mouse monoclonal Serum Amyloid A antibody (Novus Biologicals) in 1:100 dilution for 30 min. and processed with detection antibodies (DAKO REAL EnVision+ Dual Link, Single Reagents, HRP Rabbit/Mouse) for 30 min. The sections were immersed in 3.3% diaminobenzidine (Dako Company) chromogen dye and hydrogen peroxide buffer solution for 4 min. This created a brownish staining in the location of detection antibody. The background was dyed with hematoxylin, dehydrated with 2x 96% alcohol and 2x xylene, covered with aqueous resin. The immunohistochemistry visual validation procedure was carried out by an independent pathologist. Within the publication data 1 PD patient (male, age 74) and 1 control (female, age 64) have been presented as representative images.

## Results

Our previous work with skin RNA-sequencing showed a significant downregulation of *Saa-1* and *-2* expression levels in PD patients (logFC -2,75 and -1,65, respectively, Planken et al., 2017). Further tests with 86 PD patients, who met the Queen Square Brain Bank diagnostic criteria (Gibb and Lees, 1988; Lees et al., 2009) and 72 healthy matched controls were performed. *Saa-1* and *-2* gene expression levels were measured using qRT-PCR from skin, followed by measurement of SAA1/2 protein levels from serum using ELISA and finally the SAA1/2 protein levels were assayed using immunohistochemistry from PD patients’ skin.

### Gene Expression Profiling of *Saa-1* and *-2* in Skin and Blood From PD Patients

**FIGURE 1:**
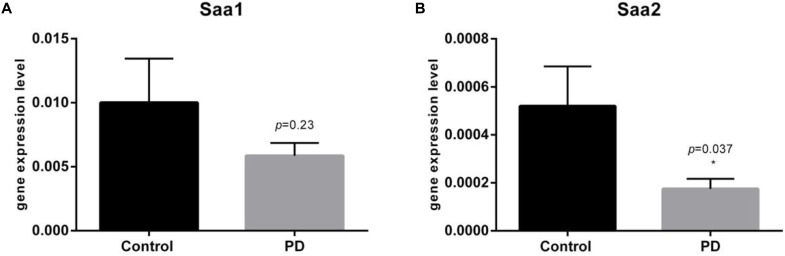
qRT-PCR on relative gene expression levels of *Saa1* **(A)** and *Saa2* **(B)** genes in the skin of 33 PD patients and 37 controls. Barplots show mean fold changes with upper percentiles displayed in 2^-ΔCT^ values. **(A)** Demonstrating not significantly (*p* = 0.25) lowered *Saa1* levels in PD patients by 1.34-fold. Mean relative gene expression for controls is 0.01001 (SEM = 0.003431) and for PD patients 0.005853 (SEM = 0.001007). **(B)** Demonstrating significantly (*p* = 0.037) lowered *Saa2* levels in PD patients by 1.68-fold. Mean relative gene expression for controls is 0.0005200 (SEM = 0.0001653) and for PD patients 0.0001749 (SEM = 4.242e-005).

The qRT-PCR analysis from 33 PD patients and 37 controls demonstrated a significant 1.68-fold downregulation of *Saa2* gene (*p* = 0.0372) and 1.34-fold downregulation of *Saa1* gene in PD patients skin, however the result was not statistically significant (*p* = 0.2469) (Figure 1).

### SAA Protein Visualization in the Skin

**FIGURE 2:**
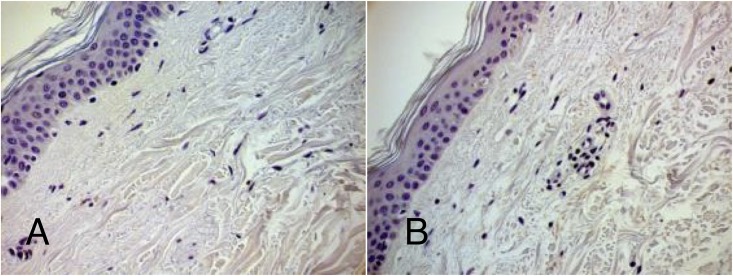
Representative IHC for SAA 1/2. Control **(A)** and PD patient **(B)**. Magnification: 40×. Dye: Hematoxylin and immunohistochemical labeling for serum amyloid alpha. Both in control and PD patient no detectable visual staining can be demonstrated.

The immunohistochemical analysis of 13 PD patients’ and 12 controls’ skins for expression of SAA 1/2 protein, demonstrated that no visually detectable protein or changes in protein levels can be observed in PD patients (Figure 2).

### ELISA From Serum

**FIGURE 3:**
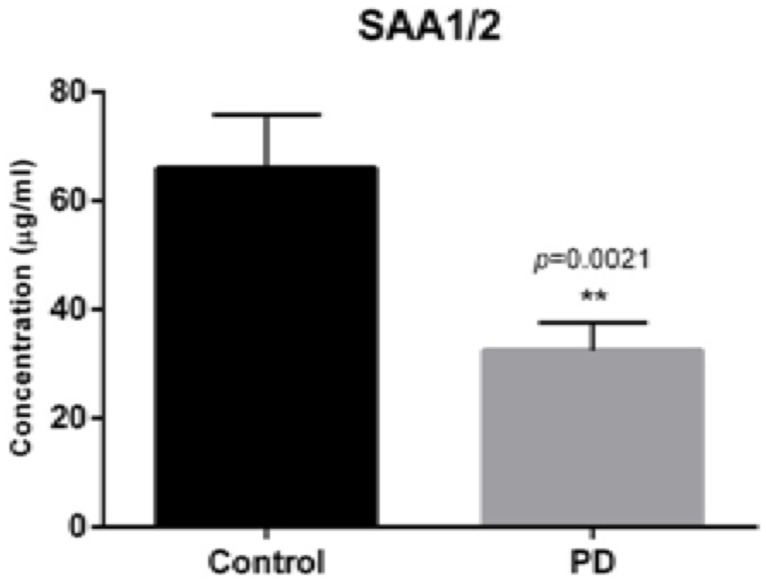
SerumELISA SAA1/2 concentration in 27 controls and 36 PD patients, respectively. Demonstrating decreased concentration of SAA1/2 by 50.9% (*p* = 0.0021). Bars show mean concentration with upper percentiles, 66.8 μg/ml for healthy controls and 32.8 μg/ml for PD patients (*p* = 0.0054).

ELISA analysis (from 36 PD patients and 27 controls) of serum for SAA 1/2 protein expression, demonstrated decreased protein concentration levels in the serum of PD patients by 50.9% compared to controls (Figure 3, *p* = 0.0021), with the mean protein concentrations being 32.8 and 66.8 μg/ml, respectively.

## Discussion

The qRT-PCR results of SAA downregulation in the skin mirrored the changes obtained in RNA-sequencing (Planken et al., 2017). *Saa-1* and *-2* RNA levels in whole-blood cells were below the detection levels using RNA-sequencing (unpublished data). In blood serum, SAA1/2 was downregulated, implying SAA1/2 is secreted into blood. In skin immunohistochemistry, SAA1/2 protein does not visualize, thus downregulation of the gene is not detectable using this method. As the quantity level for these proteins in the normal skin is physiologically low, this is to be expected. SAA is known as a marker for AA-amyloidosis and positive staining is seen only in patients suffering from chronic inflammation, certain cancers or genetic defects, in which case, the pathologic staining would be seen around basal membranes, blood vessel walls and collagenous connective tissue (James et al., 2011). In addition, Western blot analysis on the same subject samples were performed, however the levels of SAA in skin homogenates were also too low for detection (data not shown). Taken together, the findings show downregulated *Saa-1* and *-2* expression in skin and protein SAA1/2 levels in blood.

### SAA as an Immunomodulator in the Skin

SAA in the skin tissue is an autocrine modulatory protein, induced by inflammatory signals like IL-11α, TNF-α, and IL-17A and stimulating the expressions of other cytokines, interleukins and metalloproteinases on the tissue level (Patel et al., 1998; Furlaneto and Campa, 2000; Vallon et al., 2001; de Seny et al., 2013). SAA upregulates its own expression, creating an autocrine self-maintaining regulation by positive feedback (Couderc et al., 2017). In our previous work with skin RNA-sequencing a large set of genes in PD skin were downregulated, which are involved in the process of epidermal cornification and desquamation (Planken et al., 2017). The downregulation of SAA, an important autocrine immunomodulator in PD skin, might contribute to the impaired epidermal renewal processes which are clinically associated with poor wound healing in PD patients. Moreover, serum SAA has been shown to be elevated in patients of all stages of melanoma (I to IV) and is proposed as a prognostic biomarker for the disease (Findeisen et al., 2009). Different types of cancers occur less frequently in PD patients, however the risk of melanoma is about 2-fold higher than in general population (Liu et al., 2011). These findings corroborate the role of SAA within the pathomechanistic link between melanoma and PD. It would be of interest to measure the levels of keratinocyte-derived SAA in patients diagnosed with both PD and melanoma to elaborate these findings.

### SAA as an Intrinsically Disordered Protein

Intrinsically disordered proteins (IDPs) refer to proteins without fixed three-dimensional structures under physiological conditions, allowing the same polypeptide to undertake different interactions with different consequences (Zhang et al., 2013). This lack of proper fixed structure enables IDPs to interact with many ligands, but renders them vulnerable to environmental changes and causes them to misfold. IDPs are involved in neurodegenerative diseases (Uversky, 2009, 2015), which all exhibit characteristic accumulation of incorrectly folded proteins that hence form insoluble deposits (Takalo et al., 2013). SAA is an IDP (Ye et al., 2011; Colón et al., 2015) and therefore able to contribute to protein deposit formation in PD. Overexpression of IDPs, like a-synuclein, in the CNS is known to cause neurodegeneration in model organisms (Kirik and Björklund, 2003). It is suggestive that SAA acts similarly in the CNS of PD. In a mouse model of AD, systemic LPS was administered (Liu et al., 2016), which induced SAA expression that led to activation of brain microglia and to suppression of tau hyperphosphorylation, having a modifying effect on neurofibrillary tangle development. Acute SAA is shown to be elevated in human AD brains (Liang et al., 1997) and cerebrospinal fluid (Kindy et al., 1999). Neurodegenerative diseases, like AD and PD are associated with neuroinflammation, suggesting that also the levels of SAA, as one of acute phase reactants, are expected to be higher in the CNS. How SAA acts in the periphery in neurodegenerative diseases is largely unknown. We now show that SAA is downregulated in the peripheral tissues like skin and blood, which could be a compensatory effect for excessive levels of aberrant SAA in the CNS.

### Global Peripheral Downregulation of SAA Protein

Within this study we have also demonstrated significantly lowered protein levels of SAA in PD serum. This finding suggests that the downregulation of SAA proteins occurs in PD patients not only on the transcriptomic level in the skin, but more globally also in other tissues. Liver is the predominant synthesis place of SAA, from where SAA proteins are secreted into the circulation. The downregulated levels in serum indicate a reduced transcription of SAA in the liver tissue. In the skin, no SAA could be detected using immunohistochemistry and by Western blot method. This was expected, as the accumulation of misfolded SAA is known as AA-Amyloidosis and it seems to appear in the conditions of chronic inflammation with elevated serum levels of SAA. We showed, instead, downregulated levels of SAA in both skin and serum. In the normal brain tissue, SAA is not detectable, but it has been shown to be elevated in the CNS under induced inflammatory conditions and in case of Alzheimer’s disease (Liang et al., 1997; Guo et al., 2002). How SAA acts in the CNS in case of PD and whether its expression in the periphery affects it, is largely unknown. We have now characterized the downregulation of SAA in PD skin and serum and demonstrated that no visible deposits of SAA protein in the skin of PD patients can be seen.

## Ethics Statement

Research Ethics Committee of the University of Tartu 3-318 51003.

## Author Contributions

AP, LK, LT, TE, and ER carried out the laboratory work. LK-E performed patient selection and clinical analysis. KK contributed to study planning and reviewing the manuscript. SK and AP involved study planning, performed analysis of sequencing data, and wrote the manuscript. PT participated in writing and reviewing of the manuscript. All authors read and approved the final manuscript.

## Conflict of Interest Statement

The authors declare that the research was conducted in the absence of any commercial or financial relationships that could be construed as a potential conflict of interest.

## Supplementary Material

TABLE S1 Breakdown of patient and control clinical characteristics.
