# Supplementary material for: Serum Amyloid Alpha Is Downregulated in Peripheral Tissues of Parkinson’s Disease Patients
Source: Front Neurosci. 2019 Jan 29;13:13. doi: 10.3389/fnins.2019.00013 (PMC6361740; doi:10.3389/fnins.2019.00013)
Supplement: TABLE S1 — Breakdown of patient and control clinical characteristics. [file Table_1.DOCX]

|  | Subj. no | Gender | Age (yr) | | Disease onset age (yr) | Duration of disease (years) | Relatives with PDˇ | Leading symptom* | HY | SE-ADL | MMSE | MDS-UPDRS |
| --- | --- | --- | --- | --- | --- | --- | --- | --- | --- | --- | --- | --- |
| qRT-PCR PD | | | | | | | | | | | | |
|  | 1 | M | 68 | 65 | | 2,5 | 3 | 3 | 2,5 | 70 | 29 | 66 |
|  | 2 | N | 65 | 55 | | 10 | 1 | 4 | 4 | 60 | 30 | 114 |
|  | 3 | M | 73 | 65 | | 9 | 1 | 3 | 3 | 80 | 30 | 77 |
|  | 4 | M | 65 | 57 | | 9 | 1 | 1 | 1,5 | 90 | 30 | 52 |
|  | 5 | N | 80 | 63 | | 17 | 2 | 1 | 3 | 70 | 28 | 92 |
|  | 6 | M | 77 | 71 | | 6 | 1 | 1 | 2 | 95 | 24 | 42 |
|  | 7 | M | 68 | 53 | | 15 | 1 | 3 | 2 | 90 | 30 | 49 |
|  | 8 | M | 60 | 46 | | 14 | 3 | 3 | 2,5 | 80 | 28 | 83 |
|  | 9 | M | 71 | 70 | | 0,9 | 1 | 1 | 1,5 | 90 | 29 | 33 |
|  | 10 | N | 66 | 64 | | 2 | 1 | 3 | 2 | 90 | 29 | 35 |
|  | 11 | M | 54 | 46 | | 8 | 1 | 3 | 2 | 90 | 30 | 51 |
|  | 12 | N | 69 | 65 | | 4 | 1 | 1 | 1,5 | 90 | 30 | 59 |
|  | 13 | M | 72 | 63 | | 9 | 1 | 3 | 3 | 90 | 30 | 53 |
|  | 14 | M | 77 | 67 | | 10 | 1 | 1 | 4 | 60 | 27 | 104 |
|  | 15 | M | 70 | 68 | | 2 | 1 | 1 | 1,5 | 90 | 29 | 43 |
|  | 16 | M | 60 | 52 | | 8 | 1 | 2 | 2,5 | 90 | 30 | 37 |
|  | 17 | N | 68 | 61 | | 7,4 | 1 | 3 | 1,5 | 90 | 30 | 33 |
|  | 18 | M | 58 | 46 | | 12 | 3 | 2 | 3 | 80 | 29 | 102 |
|  | 19 | N | 57 | 53 | | 4 | 1 | 1 | 1,5 | 90 | 30 | 51 |
|  | 20 | N | 76 | 67 | | 9 | 1 | 1 | 3 | 80 | 30 | 85 |
|  | 21 | N | 71 | 60 | | 11 | 2 | 1 | 2,5 | 80 | 30 | 58 |
|  | 22 | N | 82 | 74 | | 9,5 | 1 | 4 | 4 | 60 | 26 | 79 |
|  | 23 | N | 58 | 47 | | 13 | 1 | 1 | 2,5 | 90 | 26 | 71 |
|  | 24 | N | 71 | 47 | | 25 | 1 | 4 | 4 | 55 | 30 | 115 |
|  | 25 | N | 66 | 60 | | 7 | 1 | 1 | 2 | 90 | 30 | 43 |
|  | 26 | N | 73 | 60 | | 14 | 2 | 3 | 2,5 | 80 | 28 | 54 |
|  | 27 | N | 77 | 68 | | 9 | 1 | 3 | 3 | 70 | 30 | 68 |
|  | 28 | N | 64 | 58 | | 6 | 3 | 2 | 2,5 | 90 | 30 | 35 |
|  | 29 | M | 74 | 63 | | 11 | 1 | 3 | 3 | 70 | 25 | 55 |
|  | 30 | M | 63 | 58 | | 3,7 | 1 | 1 | 2,5 | 90 | 30 | 40 |
|  | 31 | M | 83 | 72 | | 10 | 1 | 1 | 2,5 | 80 | 28 | 68 |
|  | 32 | N | 76 | 73 | | 2,9 | 1 | 1 | 3 | 80 | 27 | 77 |
|  | 33 | M | 67 | 64 | | 2,8 | 1 | 3 | 2 | 90 | 29 | 65 |
|  | 34 | N | 68 | 65 | | 3 | 1 | 1 | 1,5 | 100 | 30 | 26 |
|  | 35 | M | 75 | 64 | | 12 | 1 | 4 | 4 | 70 | 30 | 105 |
|  | 36 | N | 69 | 67 | | 2 | 1 | 1 | 2,5 | 80 | 29 | 72 |
|  | 37 | N | 80 | 76 | | 4,3 | 1 | 3 | 2 | 95 | 25 | 28 |
| n/% | 37 | M=18/49 |  |  | |  | 1=30/81,1  2=3/8,1  3=4/10,8 | 1=17/46  2=3/8  3=13/35  4=4/11 |  |  |  |  |
| Mean ± SD |  |  | 69.5±7.3 | 61.4±8.3 | | 8.2±5.1 |  |  | 2.5±0.8 | 82.0±11.3 | 28.8±1.7 | 62.7±24.8 |
| qRT-PCR HC |  | | | | | | | | | | | |
| n/% | 33 | M=12/36 |  | |  |  | - | - |  |  |  |  |
| Mean ± SD |  |  | 72.1±7.9 | | - | - |  |  | - | - | - | - |
| ELISA PD | | | | | | | | | | | | |
|  | 1 | M | 73 | | 65 | 9 | 1 | 3 | 3 | 80 | 30 | 77 |
|  | 2 | N | 65 | | 57 | 8 | 1 | 1 | 2,5 | 80 | 29 | 76 |
|  | 3 | M | 58 | | 48 | 10 | 1 | 3 | 4 | 60 | 30 | 110 |
|  | 4 | N | 77 | | 71 | 7,5 | 1 | 1 | 3 | 70 | 23 | 116 |
|  | 5 | N | 77 | | 68 | 9 | 1 | 3 | 3 | 70 | 30 | 68 |
|  | 6 | N | 71 | | 66 | 5 | 1 | 1 | 1,5 | 100 | 30 | 36 |
|  | 7 | N | 78 | | 7 | 1,5 | 1 | 4 | 3 | 80 | 25 | 51 |
|  | 8 | N | 76 | | 67 | 9 | 1 | - | - | - | - | - |
|  | 9 | N | 76 | | 73 | 2,9 | 1 | 1 | 3 | 80 | 27 | 77 |
|  | 10 | M | 81 | | 80 | 1,4 | 1 | 1 | 2,5 | 90 | 30 | 43 |
|  | 11 | N | 81 | | 80 | 1,7 | 1 | 1 | 3 | 75 | 23 | 94 |
|  | 12 | M | 67 | | 64 | 2,8 | 1 | 3 | 2 | 90 | 29 | 65 |
|  | 13 | N | 68 | | 65 | 3 | 1 | 1 | 1,5 | 100 | 30 | 26 |
|  | 14 | N | 71 | | 66 | 5 | 1 | 3 | 3 | 70 | 24 | 73 |
|  | 15 | N | 70 | | 67 | 2,9 | 2 | 1 | 2,5 | 80 | 29 | 83 |
|  | 16 | M | 75 | | 64 | 12 | 1 | 4 | 4 | 70 | 30 | 105 |
|  | 17 | N | 48 | | 47 | 1,1 | 1 | 3 | 1,5 | 90 | 30 | 33 |
|  | 18 | N | 69 | | 67 | 2 | 1 | 1 | 2,5 | 80 | 29 | 72 |
|  | 19 | M | 77 | | 75 | 2 | 1 | 4 | 4 | 60 | 21 | 86 |
|  | 20 | N | 68 | | 62 | 6 | 1 | 3 | 1 | 95 | 30 | 34 |
|  | 21 | M | 84 | | 79 | 4,5 | 1 | 1 | 3 | 75 | 27 | 72 |
|  | 22 | N | 77 | | 69 | 8 | 1 | 4 | 5 | 40 | 20 | 100 |
|  | 23 | N | 81 | | 77 | 3,3 | 1 | 3 | 3 | 80 | 21 | 79 |
|  | 24 | M | 60 | | 59 | 1,4 | 1 | 3 | 1,5 | 90 | 29 | 43 |
|  | 25 | N | 78 | | 60 | 18 | 2 | 4 | 4 | 50 | - | 131 |
|  | 26 | M | 70 | | 35 | 35 | 1 | 3 | 5 | 40 | 25 | 167 |
|  | 27 | N | 77 | | 73 | 4 | 1 | 4 | 3 | 60 | 27 | 73 |
|  | 28 | M | 51 | | 43 | 7,8 | 2 | 1 | 2 | 90 | 27 | 57 |
|  | 29 | N | 68 | | 63 | 4,7 | 1 | 1 | 4 | 75 | 30 | 86 |
|  | 30 | N | 79 | | 78 | 1,2 | 2 | 1 | 1 | 90 | 29 | 35 |
|  | 31 | M | 77 | | 77 | 0,9 | 1 | 1 | 2,5 | 80 | 28 | 71 |
|  | 32 | M | 73 | | 72 | 1 | 1 | 1 | 1 | 95 | 29 | 22 |
|  | 33 | N | 84 | | 83 | 1,2 | 1 | 4 | 2,5 | 80 | 29 | 64 |
|  | 34 | N | 63 | | 58 | 5 | 1 | 1 | 2,5 | 90 | 25 | 69 |
|  | 35 | N | 77 | | 75 | 2 | 1 | 1 | 2,5 | 80 | 24 | 56 |
|  | 36 | N | 70 | | 64 | 6 | 1 | 1 | 3 | 70 | 21 | 62 |
| n/% | 36 | M=12/33 |  | |  |  | 1=31/88,6  2=4/11,4  3=0 | 1=18/50  2=0  3=10/28  4=7/19 |  |  |  |  |
| Mean ± SD |  |  | 72.1±8.4 | | 66.5±10.8 | 5.7±6.3 |  |  | 2.7±1.0 | 77.3±14.9 | 27.1±3.2 | 71.2±30.7 |
| ELISA HC |  | | | | | | | | | | | |
| n/% | 27 | M=10/37 |  | |  |  |  |  |  |  |  |  |
| Mean ± SD |  |  | 72.7±9.6 | |  |  |  |  |  |  |  |  |
| Immunohistochemistry PD | | | | | | | | | | | | |
|  | 1 | M | 60 | | 46 | 14 | 3 | 3 | 2,5 | 80 | 28 | 83 |
|  | 2 | F | 82 | | 74 | 9 | 1 | 4 | 4 | 60 | 26 | 79 |
|  | 3 | F | 77 | | 68 | 9 | 1 | 3 | 3 | 70 | 30 | 68 |
|  | 4 | M | 63 | | 58 | 4 | 1 | 1 | 2,5 | 90 | 30 | 40 |
|  | 5 | F | 76 | | 73 | 3 | 1 | 1 | 3 | 80 | 27 | 77 |
|  | 6 | M | 81 | | 80 | 1 | 1 | 1 | 2,5 | 90 | 30 | 43 |
|  | 7 | M | 67 | | 64 | 3 | 1 | 3 | 2 | 90 | 29 | 65 |
|  | 8 | F | 68 | | 65 | 3 | 1 | 1 | 1,5 | 100 | 30 | 26 |
|  | 9 | M | 75 | | 64 | 11 | 1 | 4 | 4 | 70 | 30 | 105 |
|  | 10 | F | 69 | | 67 | 2 | 1 | 1 | 2,5 | 80 | 29 | 72 |
|  | 11 | F | 80 | | 76 | 4 | 1 | 3 | 2 | 95 | 25 | 28 |
|  | 12 | F | 65 | | 55 | 10 | 1 | 4 | 4 | 60 | 30 | 114 |
|  | 13 | M | 74 | | 63 | 11 | 1 | 3 | 3 | 70 | 25 | 55 |
| n/% | 13 | M=5/38,5 |  | |  |  | 1=12/92  3=1/8 | 1=5/38,5  2=0  3=5/38,5  4=3/23 |  |  |  |  |
| Mean ± SD |  |  | 72,1±7,2 | | 65,6±9,2 | 6,5±4,3 |  |  | 2,8±0,8 | 79,6±13,0 | 28,4±2,0 | 65,8±27,1 |
| Immunohistochemistry HC  n/%  Mean ± SD |  | | | | | | | | | | | |
|  | 12 | M=3/25 |  | |  |  |  |  |  |  |  |  |
|  |  | 71,8±8,6 |  | |  |  |  |  |  |  |  |  |
| Total PD |  | | | | | | | | | | | |
| n/% | 86 | M=36/41,9 |  | |  |  | 1=73/86  2=7/8  3=5/6 | 1=40/47  2=3/4  3=28/33  4=14/16 |  |  |  |  |
| Mean ± SD |  |  | 71,0±7,8 | | 64,2±9,8 | 6,9±5,6 |  |  | 2,7±0,9 | 79,7±13,2 | 28,0±2,6 | 52,4±14,1 |

*Leading clinical symptom of PD: 1- Tremor; 2 - Rigidity; 3 - Bradykinesia; 4 – Postural instability.

ˇFamilial incidence of PD: 1 - no relatives with PD, 2 - relatives with PD of 1^st^ degree, 3 - relatives with PD of 2^nd^ degree

Abbrevations: PD, Parkinson´s disease; HY, Hoehn and Yahr stage; SE-ADL, Schwab and England Activities of Daily Living Scale; MMSE, Mini Mental State Examination; MDS-UPDRS, Movement Disorders Society Unified Parkinson´s Disease Rating Scale.
